# Rectal Colonization by Carbapenemase-Producing *Enterobacterales* in a Tertiary Care Hospital in Havana, Cuba

**DOI:** 10.3390/antibiotics15010109

**Published:** 2026-01-22

**Authors:** Haiyang Yu, Yenisel Carmona, Vismayda Bouza, María Karla González, Gonzalo Estevez Torres, Valia Ramos Rodríguez, Alberto Hernández González, Nobumichi Kobayashi, Meiji Soe Aung, Patricia Ruiz-Garbajosa, Rafael Cantón, Dianelys Quiñones Pérez

**Affiliations:** 1 Henan Provincial People’s Hospital, People’s Hospital of Zhengzhou University, Zhengzhou 450003, China; 2 Tropical Medicine Institute “Pedro Kourí”, Havana 11400, Cuba; 3 “Hermanos Ameijeiras” Hospital, Havana 10348, Cuba; 4 Department of Social Medicine, Division of Hygiene, School of Medicine, Sapporo Medical University, Sapporo 060-8556, Japan; nkobayas@sapmed.ac.jp (N.K.); meijisoeaung@sapmed.ac.jp (M.S.A.); 5 Servicio de Microbiología, Hospital Universitario Ramón y Cajal, Instituto Ramón y Cajal de Investigación Sanitaria, 28034 Madrid, Spainrafael.canton@salud.madrid.org (R.C.); 6 CIBER de Enfermedades Infecciosa, Instituto de Salud Carlos III, 28029 Madrid, Spain

**Keywords:** rectal colonization, *Enterobacterales*, carbapenemases, carbapenem resistance, infection control, Cuba

## Abstract

**Introduction:** Rectal colonization by carbapenemase-producing carbapenem-resistant *Enterobacterales* (CP-CRE) is a risk factor for subsequent infections, which are associated with high mortality rates. **Methods:** A cross-sectional study was conducted. Rectal swabs were collected from 297 patients within 48 h of admission to eight high-prevalence CP-CRE hospital departments, with follow-up swabs taken weekly for up to 4 weeks. Species identification, antimicrobial susceptibility testing, and genetic detection of carbapenemases were performed. The genetic relationship among isolates was assessed using ERIC-PCR, combined with epidemiological data, to investigate subsequent infections. **Results:** Fecal carriage of CP-CRE was detected in 15.5% (46/297) of patients- All carbapenemases were metallo-betalactamases, with dominance of NDM-producing *Klebsiella pneumoniae*. NDM + VIM-producing *Escherichia coli* were also detected. Among carriers, 26.1% were colonized by two different CRE species, and 86.9% had a history of prior hospitalization. Molecular analysis revealed clonal expansion, suggesting outbreaks among colonized patients. Additionally, 17.4% (8/46) of colonized patients developed an infection, which was significantly associated with urinary catheter use (*p* = 0.040), mechanical ventilation (*p* = 0.044), and surgical procedures (*p* = 0.040). **Conclusions:** rectal colonization by CP-CRE in hospitalized patients is a serious epidemiological concern, with evidence of clonal spread and subsequent infection in colonized patients. NDM-producing *K. pneumoniae* was also predominant, detecting co-production of NDM + VIM in *E. coli*. These findings underscore the urgent need to implement epidemiological surveillance cultures to improve the prevention and control of CP-CRE infections in Cuban hospitals.

## 1. Introduction

Recent years have witnessed an accelerated global spread of highly drug-resistant microorganisms, with a notable rise in carbapenem resistance. This trend is driven by the overuse of broad-spectrum antibiotics, healthcare system overcrowding, and lapses in infection prevention and control (IPC) measures [1]. The resulting high-attributable mortality and prolonged hospital stays associated with carbapenem-resistant infections pose a severe public health and economic threat globally [2].

In response, WHO recommends member states strengthen epidemiological surveillance and research on carbapenem resistance mechanisms to inform timely IPC interventions and mitigate nosocomial transmission. Specifically, the implementation of active surveillance cultures is a cornerstone of recommended IPC bundles for multidrug-resistant organisms in high-risk settings [3].

Rectal colonization with carbapenemase-producing carbapenem-resistant *Enterobacterales* (CP-CRE) is closely linked to subsequent invasive infection and increased mortality, especially in critically ill hospitalized patients. The gastrointestinal tract serves as a major reservoir for CP-CRE, highlighting the clinical significance of identifying colonized individuals. Studies demonstrate that a significant proportion of clinical CP-CRE infections originate from prior, undetected rectal colonization [4].

Active surveillance via rectal screening for CP-CRE carriage is therefore crucial for early identification of transmission sources and providing data to optimize IPC policies. This approach has been shown to interrupt transmission chains and reduce infection rates in endemic and outbreak situations [5].

In Cuba, previous CP-CRE studies have focused on clinical isolates, with scarce data on rectal colonization. This gap is also noted in several Latin American and Caribbean countries, where surveillance of colonization prevalence remains inconsistent, hindering regional risk assessments and control strategies. This first Cuban study aims to estimate CRE colonization prevalence in a tertiary hospital, identify colonization risk factors, and evaluate progression from colonization to infection. It fills critical gaps to strengthen Cuba’s antimicrobial resistance (AMR) response efforts.

## 2. Results

### 2.1. Prevalence and Epidemiological Characteristics of Intestinal CP-CRE Colonization

During the study period, 297 patients were screened, of whom 46 (15.5%, 95% CI: 11.6–20.2%) tested positive for CP-CRE. Among these 46 carriers, 12 (26.1%, 95% CI: 14.3–41.1%) were simultaneously colonized by two different CRE species. Most colonized patients (37/46, 80.4%, 95% CI: 66.1–90.6%) were identified during the first screening (≤48 h after admission) and were classified as having “pre-admission rectal colonization”; the remaining 9 (19.6%, 95% CI: 9.4–33.9%) were identified later and classified as “hospital-acquired rectal colonization.”

Forty carriers (86.9%, 95% CI: 73.8–94.9%) had been hospitalized within the preceding year (interval: 1–9 months). Twenty (43.5%, 95% CI: 28.9–58.9%) had received prior antibiotic therapy, including third-generation cephalosporins (*n* = 8), fluoroquinolones (*n* = 7), aminoglycosides (*n* = 6), carbapenems (*n* = 3), and piperacillin-tazobactam (*n* = 1).

**Figure 1:**
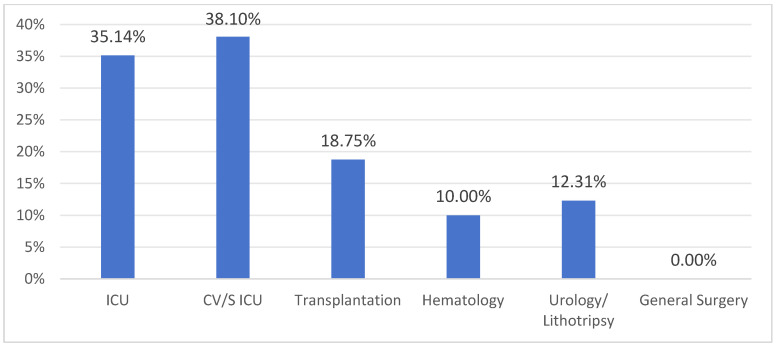
Detection rate of rectal colonization by CP-CRE by hospital department. ICU: Intensive Care Unit; CV/S ICU: Cardiovascular Surgical Intensive Care Unit.

The detection rate of CP-CRE colonization varied by hospital department (Figure 1). No colonized patients were detected in General Surgery, while rates in other departments ranged from 10.00% to 38.10%. The prevalence in Intensive Care Units (ICUs) was approximately three times as high as in other wards.

### 2.2. Microbiological Characteristics of Colonizing Isolates

A total of 58 CRE isolates were obtained from the 46 colonized patients. The most prevalent species were *Klebsiella pneumoniae* (41.4%, *n* = 24), followed by *Escherichia coli* (32.8%, *n* = 19) and *Enterobacter cloacae* (15.5%, *n* = 9). Single isolates of *Citrobacter koseri*, *C. freundii*, *Morganella morganii*, *Klebsiella ornithinolytica*, *K. aerogenes*, and *K. oxytoca* were also detected.

All isolates (*n* = 58) produced metallo-β-lactamases: 98.3% were NDM-type, 15.5% were VIM-type, and 13.8% co-produced NDM and VIM. No other carbapenemase types were detected. VIM-type carbapenemase was found exclusively in *E. coli*.

Among the 12 patients with dual-species colonization, eight carried different species harboring the same carbapenemase gene (NDM), while four carried species with different carbapenemase profiles (NDM and/or VIM).

Antimicrobial susceptibility testing revealed high resistance rates to most agents (Table 1). Resistance to colistin was observed in 25.9% of isolates. Colistin, amikacin, and fosfomycin retained the highest in vitro activity. Notably, 29.3% (*n* = 17) of isolates had a meropenem MIC of 8 µg/mL (intermediate susceptibility), while the remainder had an MIC ≥ 16 µg/mL.

### 2.3. ERIC-PCR Genotyping Analysis

**Figure 2:**
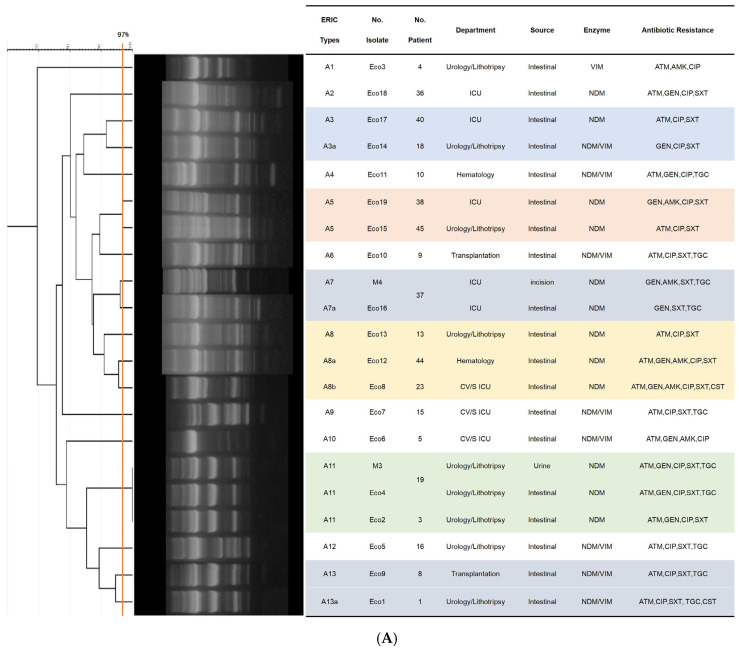

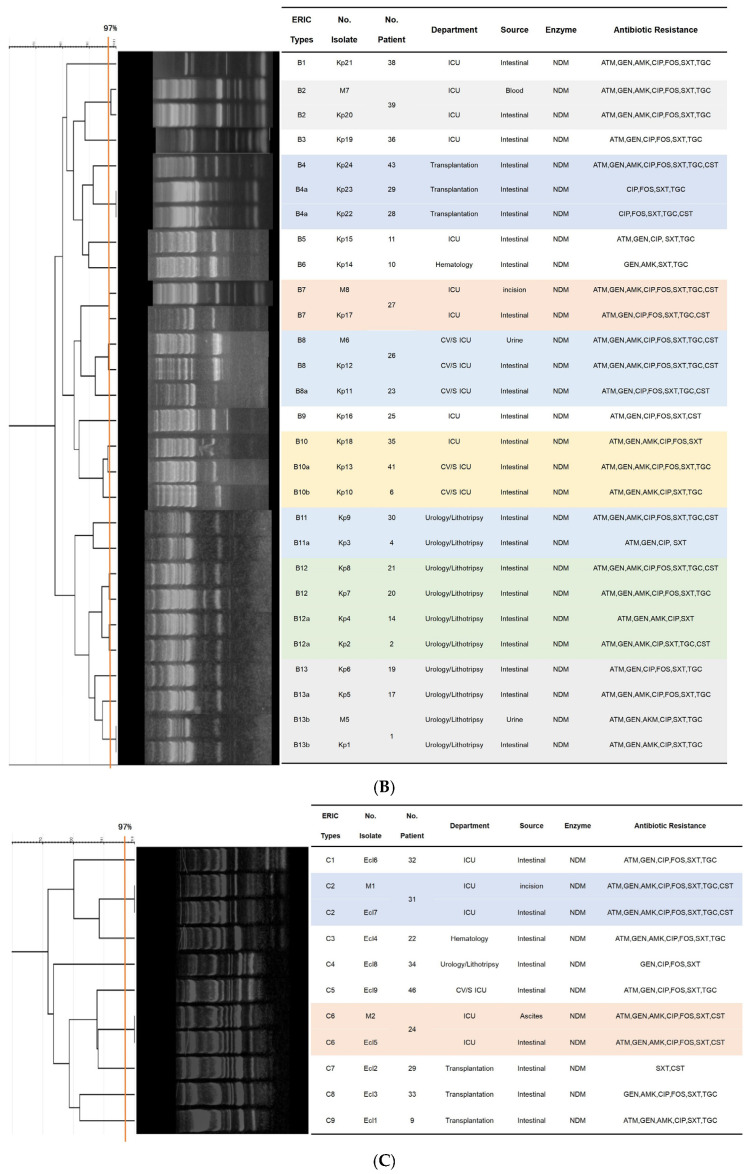
Dendrograms based on ERIC-PCR fingerprints. (**A**) 21 *E. coli* isolates; (**B**) 28 *K. pneumoniae* isolates; (**C**) 11 *E. cloacae* isolates. Note: Isolates labeled “M” indicate clinical infection isolates. Clusters (≥80% similarity), clonal variants (≥90% similarity), isolate identical (≥97% similarity) are indicated.

ERIC-PCR typing was performed on 60 isolates, which included 52 colonizing and 8 clinical strains distributed across three species: *E. coli* (*n* = 21), *K. pneumoniae* (*n* = 28) and *E. cloacae* (*n* = 11). The 21 *E. coli* strains were grouped into thirteen ERIC types (DNA fingerprint similarity ≥ 90%), comprising 15 main clusters (similarity ≥ 80%) and 3 singletons (similarity < 80%) in Figure 2A. Two clinical isolates (M3, M4) were genetically identical (Eco4) or closely related (Eco6) to the intestinal colonizing isolates from the corresponding patient, yet exhibited distinct antimicrobial resistance profiles. Four ERIC types (A3, A5, A8, A13) identified from colonizing isolates showed genetic variations and were distributed across multiple hospital departments.

The 28 *K. pneumoniae* strains were grouped into twelve ERIC types, consisting of 25 clusters in Figure 2B. Of the four clinical isolates, three (M5, M6, M7) exhibited identical genotypes (Kp1, Kp12, Kp20) and matching resistance profiles compared to their colonizing counterparts, while only one pair (M8 and Kp17) displayed a divergent resistance profile. Six ERIC types derived from colonizing samples (B4, B8, B10, B11, B12, B13) showed genetic variation, with type B10 and its variants being detected across different departments.

Analysis of the 11 *E. cloacae* strains identified nine ERIC types, comprising 7 clusters and one singletons in Figure 2C. Two isolate pairs—M1 and Ecl7, M2 and Ecl5—shared identical genotypes and matching resistance profiles; each pair comprised both colonizing and clinical isolates obtained from the same patient. All remaining colonizing isolates exhibited less than 90% genetic similarity to one another.

### 2.4. Infection Following CP-CRE Colonization

Based on ERIC-PCR analysis, clinical isolates from eight patients were identical or closely related to their corresponding colonizing isolates, confirming infection originating from prior colonization. Infections included urinary tract infections (*n* = 2), surgical site infections (*n* = 4), bacteremia (*n* = 1), and intra-abdominal infection (*n* = 1).

Univariate analysis identified urinary catheter use (PR: 1.58, 95% CI: 1.24–2.02; *p* = 0.040), mechanical ventilation (PR: 2.64, 95% CI: 1.21–5.78; *p* = 0.044), and surgical procedures (PR: 1.58, 95% CI: 1.24–2.02; *p* = 0.040) as significant risk factors for progression from colonization to infection (Table 2). A multivariate analysis was not performed due to the limited number of infection events (*n* = 8), which would preclude a stable model.

## 3. Discussion

The transmission of CP-CRE in healthcare settings is often facilitated by inadequate control measures targeting fecal carriers. Active surveillance cultures and molecular typing are critical tools for controlling nosocomial infections. However, such surveillance for carbapenemase-producing Gram-negative bacilli has not been routinely implemented in Cuban hospitals.

This study reports an overall intestinal CP-CRE colonization rate of 15.5% in a Cuban tertiary hospital, with rates exceeding 35% in ICUs. This prevalence aligns with reports from other CRE-endemic regions but with slightly lower prevalence: 17.2% in Ethiopia (2023) [6], 23.7% in a Spanish multicenter study (2024) [7], 10.84% in China (2024) [8], 28.2% in Iran (2022) [9], and 26% in an Argentine ICU (2022) [10].

Notably, 26.1% of colonized patients carried two different CRE species, a phenomenon also reported in Spain where 28.6% of patients had co-colonization with strains producing different carbapenemases [11]. The rising prevalence of CRE in Cuba likely increases the risk of multi-species colonization, highlighting an urgent need to strengthen Infection Control Programs (ICPs).

Most carriers (86.9%) had a recent hospitalization history, and 43.5% had received prior antibiotics. Colonization rates varied widely by department (0–38.1%), indicating that carrier status is influenced not only by individual risk factors but also by the local epidemiological context. Serial screening increased detection, with an 18.9% higher yield in the second week compared to the first, suggesting that continuous surveillance may be cost-beneficial.

CP-CRE colonization is a well-established risk factor for subsequent infection. A meta-analysis estimated a 16.5% risk of infection among colonized patients [12], consistent with our finding of 17.4% (8/46). Identified risk factors for progression to infection—urinary catheter, mechanical ventilation, and surgery—align with and extend previous reports focusing on ICU stay, central catheters, and broad-spectrum antibiotic exposure [13]. The duration of antibiotic exposure, a known risk factor for resistance selection [14], should be considered in future studies with larger cohorts.

Furthermore, it is notable that among the 8 patients who progressed from colonization to infection, half (4/8) developed surgical site infections (SSIs). This high proportion suggests that, in addition to the inherent breach of physical barriers during surgery, deficiencies in postoperative infection control likely play a critical role. CP-CRE colonizing the patient’s skin or gastrointestinal tract could be transferred to the surgical site via healthcare workers’ hands or contaminated environments during wound care—an exogenous transmission route supported by evidence associating poor hand hygiene compliance with increased SSI risk [15]. Concurrently, the postoperative immunosuppressed state induced by surgical stress may facilitate the establishment of infection even from a low inoculum [16]. Therefore, infection prevention should extend beyond the operating room, incorporating strict contact precautions, enhanced aseptic wound management, and attention to patients’ immune status to systematically interrupt the chain of transmission.

Remarkably, only metallo-β-lactamases (NDM and VIM) were detected, with NDM being predominant. This reflects the shifting epidemiology in Cuba, where KPC was first reported in 2011 [17], followed by a rapid increase in NDM [18]. Local hospital data also show a rise in metallo-β-lactamases since 2013 [19]. The co-production of NDM and VIM in eight *E. coli* isolates is concerning. Dual carbapenemase production is increasingly reported under carbapenem selective pressure [20,21]. VIM, first described in *Pseudomonas aeruginosa* in Italy, has a global prevalence of about 2% in *E. coli* [22] and has been associated with the pandemic clone ST131, which is prevalent in Cuba [23,24].

The finding that 66.7% of patients with dual-species colonization carried NDM in both species suggests possible horizontal transfer of mobile genetic elements between different *Enterobacterales* [25], as recently illustrated by reports of different species harboring multiple carbapenemase genes on a single plasmid [26].

Molecular typing revealed clonal expansion in *E. coli* and *K. pneumoniae* isolates, suggestive of intra-hospital outbreaks. Although ERIC-PCR yields valuable insights, its resolution is inferior to whole-genome sequencing (WGS)—the gold standard for confirming transmission links [27]. Thus, our conclusions regarding outbreaks are presented as supportive evidence, with full consideration of this methodological limitation. Inadequate environmental hygiene (e.g., contaminated sinks, toilets) and suboptimal hand hygiene compliance among healthcare workers likely facilitate the intra-hospital persistence and transmission of CP-CRE [28]. Furthermore, the pathogenic impact of these circulating clones may be compounded by virulence traits that were not characterized in this study. Successful hospital-adapted strains often carry a combination of resistance and virulence determinants, such as adhesins, siderophores, and biofilm-forming capabilities that can enhance tissue invasion and immune evasion. For example, the pandemic *E. coli* ST131 lineage, previously reported in Cuba [24], exemplifies such a dual resistance-virulence profile. Future studies incorporating virulence profiling would help clarify whether clonal expansion is driven not only by efficient transmission but also by selection of strains with heightened pathogenic potential, an insight that could refine risk stratification and prevention strategies.

### Study Limitations

This study has several limitations. It was conducted at a single center, which impair to make conclusions about this problem in Cuba. The classification of colonization detected within 48 h as “pre-admission” may not fully exclude very early hospital acquisition despite taking into account the patient’s previous hospitalization in the last 6 months. Risk factor analysis was limited to univariate methods due to the small number of infection events; multivariate analysis would be valuable in larger studies.

Additionally, in this study, the ERIC-PCR method was selected as the primary molecular typing tool due to its practical suitability within the research context, particularly considering the resource constraints typical of many low- and middle-income countries. ERIC-PCR is a well-established, cost-effective, and technically accessible technique that provides a reliable first-level assessment of bacterial relatedness. Its ability to rapidly screen a large number of *Enterobacterales* isolates and identify clusters of identical or highly similar profiles made it a pragmatic choice for initial outbreak detection. The identification of clonal expansion among CP-CRE isolates using this method offered valuable, actionable evidence suggestive of intra-hospital transmission, which is crucial for prompting immediate infection control reviews and interventions. However, the limitations of this approach are fully acknowledged. ERIC-PCR has a lower discriminatory power and genomic resolution compared to Whole-Genome Sequencing (WGS), the contemporary gold standard for confirming transmission links. While ERIC-PCR can effectively demonstrate clonality, WGS can distinguish between highly related strains, precisely identify transmission routes by detecting minor genetic variations, and confirm the directionality of spread. Furthermore, ERIC-PCR does not provide the comprehensive data on resistance genes, plasmids, and virulence factors that WGS yields. Consequently, while the clonal patterns we observed strongly support the likelihood of outbreaks, they are presented as supportive epidemiological evidence rather than definitive proof of direct transmission chains. The conclusions are therefore framed with this methodological constraint in mind, highlighting the need for WGS implementation where feasible to strengthen future surveillance and outbreak investigations in similar settings [27].

## 4. Materials and Methods

### 4.1. Study Design and Population

**Figure 3:**
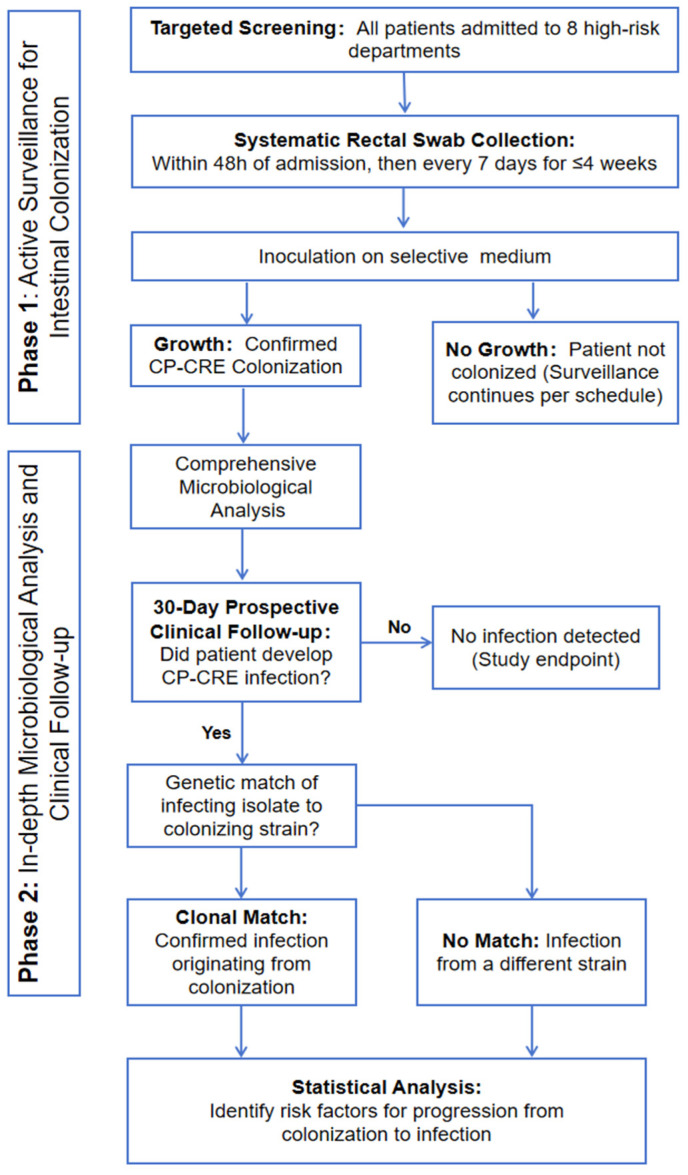
Flowchart of the study design.

A cross-sectional study with longitudinal follow-up was conducted over 12 weeks (November 2021–February 2022) at a tertiary care hospital in Havana, Cuba. The study design comprises two sequential phases: (1) Active Surveillance for Rectal Colonization and (2) In-depth Microbiological Analysis and Clinical Follow-up (Figure 3).

Phase 1: A targeted active screening program was implemented in eight clinical departments with high detection rates. All admitted patients underwent systematic rectal swab surveillance within 48 h of admission and weekly thereafter (maximum duration: 4 weeks). Swabs were cultured on selective chromogenic agar to specifically identify patients colonized with CP-CRE.

Phase 2: All CP-CRE isolates from colonized patients underwent comprehensive microbiological analysis, including species identification, carbapenemase confirmation, antimicrobial susceptibility testing, and molecular typing (e.g., ERIC-PCR) to assess clonality. Colonized patients were then prospectively followed for 30 days to monitor for the development of any subsequent CP-CRE infection (e.g., bacteremia, pneumonia, UTI). For patients who developed an infection, the infecting isolate was genetically compared to their initial colonizing strain to confirm a clonal link. Finally, statistical analysis was performed on collected clinical and demographic data to identify risk factors associated with the progression from colonization to active infection.

Inclusion Criteria: Age ≥ 18 years; New admission to one of the eight designated high-risk surveillance departments during the study period; An anticipated length of hospital stay ≥ 48 h (to ensure completion of admission screening); Provision of informed consent by the patient or their legal guardian.

Exclusion Criteria: Patients with a known status of CP-CRE colonization or infection upon admission (the study aims to detect new acquisitions); Patients with an expected survival or hospital stay of less than 48 h; Patients already hospitalized in a surveillance department for more than 48 h prior to study initiation (to avoid including prevalent colonization cases); Patients for whom rectal swab collection was anatomically or otherwise not feasible; Patients who explicitly declined to participate in any form of infection control surveillance upon admission.

Follow-up Termination Criteria: Patient discharge or transfer to another hospital; Patient death; Patient or family request to withdraw from the study; Completion of the 30-day clinical follow-up observation period.

### 4.2. Screening for Intestinal CP-CRE Carriage

Rectal swabs were collected from all participants within 48 h of hospital admission—a time threshold endorsed by the U.S. Centers for Disease Control and Prevention (CDC) as the most widely accepted standard in clinical research for differentiating pre-admission from hospital-acquired colonization and infection. Subsequent swab collections were performed weekly for a maximum of 4 weeks or until patient discharge, whichever occurred first. Swab specimens were immediately inoculated onto chromogenic mSuperCARBA^TM^ agar plates (CHROMagar^TM^, Paris, France) and incubated aerobically at 37 ± 2 °C for 48 h. Bacterial species identification was conducted using standardized microbiological protocols in accordance with the operational manual of the National Reference Laboratory for Healthcare-Associated Infections (LNR-IAAS, Institute of Public Health [IPK], Cuba).

For patients who developed clinical manifestations consistent with infection during the study period, targeted clinical samples (e.g., blood, urine, wound exudate) were collected and processed for bacterial culture. When initial phenotypic identification confirmed that the infecting pathogen matched the colonizing bacterial species, both the clinical isolate (from the infection site) and the corresponding colonizing isolate (from rectal swabs) were preserved for subsequent genetic comparison.

### 4.3. Antimicrobial Susceptibility Testing and Carbapenemase Detection

Antimicrobial susceptibility testing (AST) was performed using the Vitek2 system (bioMerieux, Marcy-l’Étoile, France) to different antibiotics included in the card VITEK^®^ 2 AST-245. Susceptibility to amikacin and tigecycline was determined by Etest (bioMerieux), and to colistin by the broth disk elution method [29]. AST results were interpreted per CLSI 2021 guidelines and EUCAST 2022 for tigecycline [30,31]. *E. coli* ATCC 25922 served as the quality control strain.

Carbapenemase genes (*bla*_IMP_, *bla*_VIM_, *bla*_NDM_, *bla*_KPC_, *bla*_OXA-48-like_) were detected by multiplex PCR as described previously [32].

### 4.4. Molecular Typing by ERIC-PCR

Clonality of *K. pneumoniae*, *E. coli* and *E. cloacae* isolates (with emphasis on colonizing/infecting pairs) was assessed by ERIC-PCR using primers ERIC1 (5′-ATGTAAGCTCCTGGGGATTCA-3′) and ERIC2 (5′-AAGTAAGTGACTGGGGTGAGAGCG-3′) [33]. Band patterns were analyzed with GelJ v1.0 software. Dendrograms were generated using the UPGMA method and Dice coefficient (5% tolerance). Isolates with ≥80% similarity were considered clustered; ≥90% indicated close relatedness; >97% indicated identity [34].

### 4.5. Data Collection and Statistical Analysis

Epidemiological and clinical data were collected retrospectively from medical records. Risk factors present before a positive screening (e.g., prior hospitalization, antibiotics) and after (e.g., device use, surgery) were recorded. A summary of patient characteristics is provided in Supplementary Table S1.

Categorical data are expressed as percentages. Univariate analysis employed the chi-square or Fisher’s exact test. Prevalence ratios (PR) with 95% confidence intervals were calculated to assess risk factors for infection progression. EPIDAT 3.1 software was used for analysis. A *p*-value ≤ 0.05 was considered significant. Assumptions for statistical tests were verified.

## 5. Conclusions

This study reports a high prevalence of intestinal CP-CRE colonization (15.5%) in a Cuban tertiary care hospital, with potential clonal outbreaks and progression to infection in 17.4% of carriers. NDM-producing *K. pneumoniae* is the predominant strain, and co-production of NDM + VIM is reported for the first time in Cuban *E. coli*. Urinary catheter use, mechanical ventilation, and surgical procedures are risk factors for infection. The findings highlight the need to strengthen infection prevention and control measures, implement systematic epidemiological surveillance, and optimize antibiotic stewardship in Cuban hospitals. Future studies should use WGS for more accurate molecular surveillance and include larger, multicenter samples to improve generalizability.

## Figures and Tables

**Table 1:** Antimicrobial resistance rates (%) of CP-CRE isolates (*n* = 58) from fecal carriers.

Antibiotic ^1^	*K. pneumoniae* (*n* = 24)	*E. coli* (*n* = 19)	*E. cloacae* (*n* = 9)	Other ^2^ (*n* = 6)	Total (*n* = 58)
AMS	100.0	100.0	100.0	100.0	100.0
TZP	100.0	100.0	100.0	100.0	100.0
CAZ	100.0	100.0	100.0	100.0	100.0
FEP	100.0	100.0	100.0	100.0	100.0
ATM	87.5	84.2	66.7	66.7	81.0
IMP	100.0	100.0	100.0	100.0	100.0
MEM	100.0	100.0	100.0	100.0	100.0
GEN	91.7	42.1	88.9	100.0	75.9
AMK	62.5	31.6	55.6	100.0	55.2
CIP	95.8	94.7	88.9	83.3	93.1
FOS	79.2	0.0	77.8	83.3	50.0
SXT	100.0	84.2	88.9	83.3	91.4
TGC	87.5	36.8	66.7	66.7	65.5
CST ^3^	37.5	10.5	33.3	20.0	25.9

^1^ Abbreviations: AMS, ampicillin-sulbactam; TZP, piperacillin-tazobactam; CAZ, ceftazidime; FEP, cefepime; ATM, aztreonam; IMP, imipenem; MEM, meropenem; GEN, gentamicin; AMK, amikacin; CIP, ciprofloxacin; FOS, fosfomycin; SXT, trimethoprim-sulfamethoxazole; TGC, tigecycline; CST, colistin. ^2^ Other species: *K. ornithinolytica*, *K. aerogenes*, *K. oxytoca*, *C. koseri*, *C. freundii*, *M. morganii*. ^3^ *M. morganii* is intrinsically resistant to colistin; its result is excluded from the total calculation for CST.

**Table 2:** Univariate analysis of risk factors for progression from CP-CRE colonization to infection.

Factors	Colonization -Infection (*n* = 8)	Colonization Only (*n* = 38)	Prevalence Ratio (95% CI)	*p*
Central Venous Catheter	3 (37.5%)	10 (26.3%)	1.52 (0.42–5.47)	0.403
Urinary Catheter	8 (100%)	24 (63.2%)	7.73 (0.48–125.32)	0.040
Nasogastric Tube	1 (12.5%)	3 (7.9%)	1.50 (0.24–9.32)	0.548
Mechanical Ventilation	5 (62.5%)	9 (23.7%)	3.81 (1.05–13.79)	0.044
Surgery	8 (100%)	24 (63.2%)	7.73 (0.48–125.32)	0.040

Footnote: Used Fisher’s exact test due to small sample sizes. *p*-values < 0.05 were considered statistically significant.

## Data Availability

The datasets generated during and/or analyzed during the current study are available from the corresponding author on reasonable request.

## Supplementary Materials

The following supporting information can be downloaded at: https://www.mdpi.com/article/10.3390/antibiotics15010109/s1, Table S1: Epidemiological and clinical data of screened patients.
