# Supplementary material for: Rectal Colonization by Carbapenemase-Producing Enterobacterales in a Tertiary Care Hospital in Havana, Cuba
Source: Antibiotics (Basel). 2026 Jan 22;15(1):109. doi: 10.3390/antibiotics15010109 (PMC12837422; doi:10.3390/antibiotics15010109)
Supplement: Supplementary file 1 [file antibiotics-15-00109-s001.zip › antibiotics-4094487-supplementary.pdf]

Table S1:Epidemiological and clinical data of screened patients

Characteristic	Total (n=297)	Colonized (n=46)	Non-colonized (n=251)
Age (mean ± SD)	56.3±17.2	58.7±16.5	55.8±17.3
Gender (male, n%)	165 (55.6%)	26 (56.5%)	139 (56.7%)
Department (n%)			
ICU	37 (12.5%)	13 (28.3%)	24 (9.6%)
CV/S ICU	21 (7.1%)	8 (17.4%)	13 (5.2%)
Transplantation	32 (10.8%)	6 (13.0%)	26 (10.4%)
Hematology	30 (10.1%)	3 (6.5%)	27 (10.8%)
Urology/Lithotripsy	130 (43.8%)	16 (34.8%)	114 (45.4%)
General Surgery	47 (15.8%)	0 (0.0%)	47 (18.7%)
Prior hospitalization (n%)	114 (38.3%)	40 (86.9%)	74 (29.5%)
Prior antibiotic use (n%)	98 (33.0%)	20 (43.5%)	78 (31.1%)
Invasive procedures (n%)			
Central venous catheter	62（20.9%）	13（28.3%）	49（19.5%）
Urinary catheter	156（52.5%）	32（69.6%）	124（49.4%）
Nasogastric Tube	47 (15.8%)	4 (8.7%)	43 (17.1%)
Mechanical ventilation	48 (16.2%)	14 (30.4%)	34 (13.5%)
Surgery	162 (54.5%)	32 (69.6%)	130 (51.8%)
